# Combination of dual serum fluorescence, AFP and hepatic function tests is valuable to identify HCC in AFP-elevated liver diseases

**DOI:** 10.18632/oncotarget.22050

**Published:** 2017-10-25

**Authors:** Ting Wang, Kun-He Zhang, Piao-Ping Hu, Qin-Si Wan, Fang-Li Han, Jian-Ming Zhou, De-Qiang Huang, Nong-Hua Lv

**Affiliations:** ^1^ Department of Gastroenterology, The First Affiliated Hospital of Nanchang University, Jiangxi Institute of Gastroenterology and Hepatology, Nanchang 330006, China

**Keywords:** hepatocellular carcinoma, AFP-elevated liver diseases, dual serum fluorescence, hepatic function tests, diagnostic model

## Abstract

Serum alpha-fetoprotein (AFP) levels elevated in benign liver diseases (BLD) represent a challenge in hepatocellular carcinoma (HCC) diagnosis. The present study aimed to develop a simple method to identify HCC in AFP-elevated liver diseases based on combining serum fluorescence and general clinical data. Serum specimens and clinical data were collected from 201 HCC and 117 BLD (41 liver cirrhosis, 76 chronic hepatitis) patients with abnormal serum AFP levels. Dual serum fluorescence (autofluorescence and cell-free DNA-related fluorescence) intensities were sequentially measured and expressed as 6 fluorescence indicators. The diagnostic value of these fluorescence and clinical data were evaluated alone and in combination by the area under receiver operating characteristic curve (AUROC). All fluorescence indicators significantly differed between HCC and BLD and some of them were more valuable for diagnosing HCC than AFP (AUROC 0.782–0.801 vs. 0.752). The diagnostic model established with fluorescence indicators, AFP, hepatic function tests and age showed that AUROC, sensitivity, specificity and accuracy were 0.958 (95% CI 0.936–0.979), 92.0%, 88.9% and 92.3%, respectively, and positive rates in AFP-negative, early and small HCCs were 73.8%, 81.6% and 74.3%, respectively. In conclusion, the combination of dual serum fluorescence, AFP, hepatic function tests and age is simple and valuable for identifying HCC in serum AFP-elevated liver diseases.

## INTRODUCTION

Hepatocellular carcinoma (HCC) is one of the most common malignant tumors worldwide. In 2015, there were 854,000 incident cases and 810,000 deaths of liver cancer globally, ranked sixth for cancer incidence and fourth for cancer death [1]. In the United States, the incidence and mortality of liver cancer have been increasing for decades, and the 5-year survival rates are 31%, 11% and 3% when diagnosed at localized, regional and distant stages, respectively [2]. Improving the survival rate in HCC patients depends on early diagnosis and treatment.

Alpha-fetoprotein (AFP) has been the most widely used serum biomarker for HCC diagnosis, but the elevated serum AFP levels (>7 ng/ml) are also frequently observed in some patients with benign liver diseases (BLD), such as liver cirrhosis (LC) and chronic hepatitis (CH) [3]. Therefore, it should be determined whether HCC is present when a patient with liver disease exhibits serum AFP elevation, especially low-level elevation (AFP < 400 ng/ml) [4]. However, the current diagnostic methods seem to be unsatisfactory for differentiating HCC from BLD in clinical practice. The areas under the receiver operating characteristic curve (AUROC) for diagnosing HCC patients with cirrhosis was 0.754 for AFP, 0.701 for protein induced by vitamin K absence or antagonist-II (PIVKA-II), 0.670 for the Lens culinaris agglutinin-reactive fraction of AFP (AFP-L3), and 0.773 for combination of the three serum biomarkers [5]. Liver cirrhosis is a major risk factor for HCC occurrence given that approximately 80–90% of HCC cases have a background of cirrhosis [6]. It may be a challenge for medical imaging to distinguish HCC foci from cirrhotic nodules. The sensitivities and specificities of computed tomography (CT), magnetic resonance imaging (MRI) and contrast-enhanced ultrasound (CEUS) for prospectively differentiating small HCC (<2 cm) from LC were 53–62% and 91–100%, respectively [7, 8]. Although histopathology is gold standard for HCC diagnosis, ultrasound-guided fine needle aspiration has only a negative predictive value of approximately 60% for differentiating HCC from BLD with serum AFP levels ≤200 ng/ml and possible serious complications (biliary peritonitis, hemorrhage and implantation metastasis) [9].

Serum-based detection is simple, practical, easily repeated and minimally invasive. Serum autofluorescence is a potential biomarker for liver diseases [10], with an accuracy of 85% for diagnosing liver cancer [11]. Cell-free circulating DNA (cfDNA) in serum has been reported as a new tumor marker based on increases in various malignancies, including HCC [12]. The AUROCs of cfDNA alone and combined with AFP for diagnosing HCC were 0.87 and 0.96, respectively [13]. Previously, we developed a simple and robust approach for noninvasively diagnosing primary hepatic carcinomas (PHC) using a combination of serum autofluorescence and cfDNA-related fluorescence sequentially detected with AUROCs of 0.773 and 0.799 for distinguishing PHC from LC and CH, respectively, and these diagnostic values are improved by combining the two types of serum fluorescence with AFP, hepatic function tests and blood cell analyses with AUROCs of 0.916 and 0.945 [14].

In the present study, we sought to develop a method to identify HCC in the liver diseases with elevated serum AFP levels. We measured serum autofluorescence and cfDNA fluorescence in HCC, LC and CH patients with abnormal serum AFP levels (>7 ng/ml) using a conventional real-time PCR system and evaluated the values of both types of serum fluorescence alone and combined with AFP, hepatic function tests and age for differentiating HCC from BLD.

## RESULTS

### Demographic and clinical data of subjects

A total of 318 patients were enrolled in this study, including 201 HCC cases and 117 BLD cases (41 LC and 76 CH). The demographic and clinical characteristics of these patients are presented in Table 1.

**Table 1:** Demographic and clinical characteristics of the patients with AFP-elevated liver diseases

	HCC (*n* = 201)	LC (*n* = 41)	CH (*n* = 76)	*P*
Age (mean ± SD, years)	51.3 ± 12.7	50.2 ± 11.5	39.5 ± 11.6^**^	<0.001^a^
Gender [*n* (%)]				
Male	176 (87.6)	33 (80.5)	60 (78.9)	0.154^b^
Female	25 (12.4)	8 (19.5)	16 (21.1)	
Etiology [*n* (%)]				
HBV	181 (90.0)	36 (87.8)	73 (96.1)	0.189^b^
HCV	0 (0.0)	1 (2.4)	3 (3.9)	0.023^c^
Alcohol	3 (1.5)	2 (4.9)	0 (0.0)	0.113^c^
Mixed	6 (3.0)	1 (2.4)	0 (0.0)	0.307^c^
Unknown	11 (5.5)	1 (2.4)	0 (0.0)	0.066^c^
AFP (μg/L)	643.2 ± 542.6	63.7 ± 120.4^**^	142.1 ± 241.1^**^	<0.001^a^
Hepatic function test (mean ± SD)				
ALT (U/L)	64.9 ± 109.7	206.0 ± 463.5	249.6 ± 241.9^**^	<0.001^a^
AST (U/L)	101.8 ± 158.7	181.4 ± 286.3	163.0 ± 177.1^*^	0.006^a^
TBIL (μmol/L)	29.0 ± 45.2	110.6 ± 143.9^**^	101.0 ± 118.1^**^	<0.001^a^
DBIL (μmol/L)	17.3 ± 33.0	66.7 ± 90.4^**^	68.8 ± 85.4^**^	<0.001^a^
GGT (U/L)	148.3 ± 158.6	73.4 ± 65.1^**^	113.8 ± 68.6^*^	0.002^a^
ALP (U/L)	164.3 ± 162.4	131.8 ± 65.4	126.4 ± 41.3^**^	0.066^a^
TP (g/L)	65.1 ± 7.3	62.1 ± 8.8	63.9 ± 7.6	0.053^a^
ALB (g/L)	35.6 ± 5.6	30.8 ± 3.9^**^	36.2 ± 5.4	<0.001^a^
GLB (g/L)	29.5 ± 6.2	31.3 ± 8.1	27.7 ± 6.0	<0.011^a^
Child-Pugh grade [*n* (%)]				
A	162 (80.6)	11 (26.8)	30 (39.5)	<0.001^b^
B	25 (12.4)	8 (19.5)	29 (38.2)	
C	14 (7.0)	22 (53.7)	17 (22.4)	

^a^One-way ANOVA test; ^b^Pearson's Chi-squared test; ^c^Fisher's exact test. ^*^P < 0.05, ^**^P < 0.01, compared with HCC by multiple comparisons in one-way ANOVA. HCC: hepatocellular carcinoma; LC: liver cirrhosis; CH: chronic hepatitis; HBV: hepatitis B virus; HCV: hepatitis C virus; AFP: alpha-fetoprotein; ALT: alanine aminotransaminase; AST: aspartate aminotransaminase; TBIL: total serum bilirubin; DBIL: direct serum bilirubin; GGT: gamma-glutamyl transferase; ALP: alkaline phosphatase; TP: total serum protein; ALB: serum albumin; GLB: serum gamma-globins.

### The fluorescence intensity measured in serum specimens

**Figure 1:**
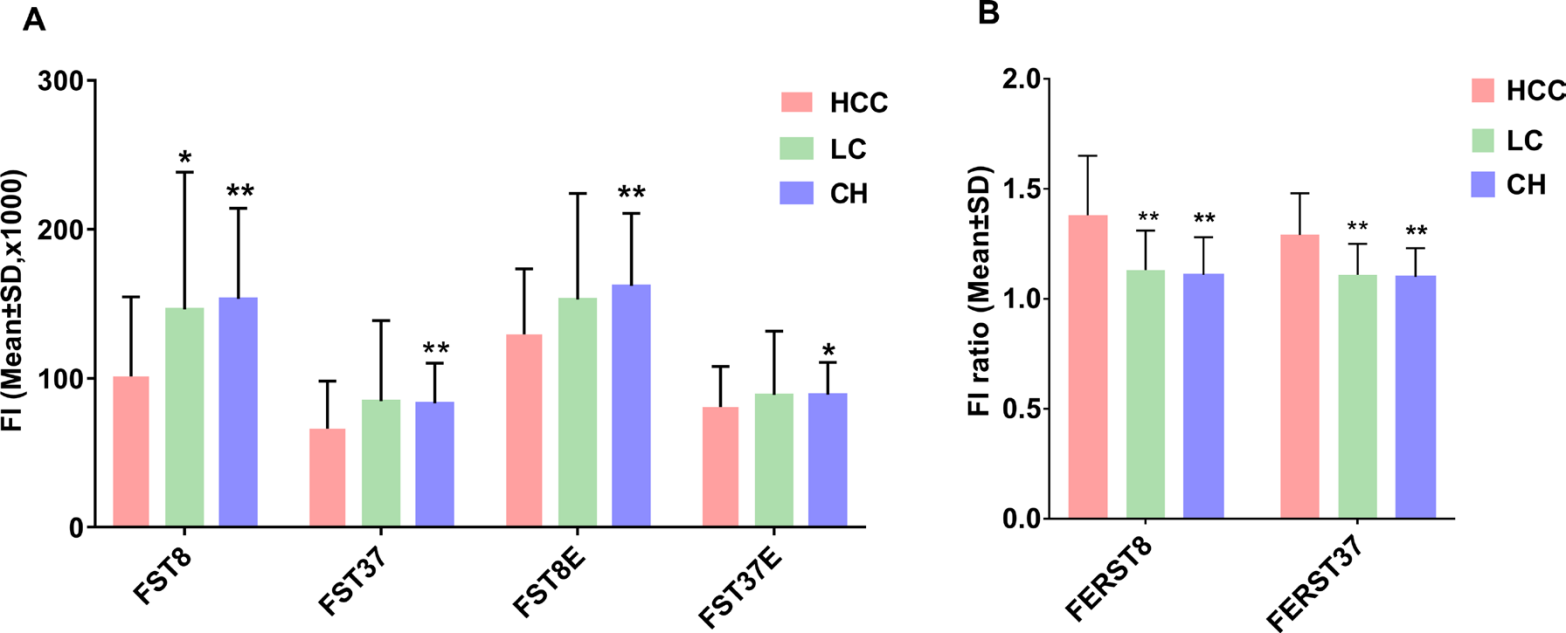
Serum fluorescence intensity results in patients with AFP-elevated liver diseases (**A**) Fluorescence intensities of four serum fluorescence indicators. (**B**) Fluorescence intensities ratio of two derived serum fluorescence indicators. ^*^P < 0.05, ^**^P < 0.01, compared with the HCC group by multiple comparisons (Dunnett’s T3 test) in one-way ANOVA. FI: fluorescence intensity; HCC: hepatocellular carcinoma, LC: liver cirrhosis; CH: chronic hepatitis. The names of fluorescence intensity indicators are abbreviations of fluorescence intensity (F) of the serum sample (S) detected at a temperature of 8°C (T8) or 37°C (T37) in the presence (E) or absence of the dsDNA dye EvaGreen or their ratio (ER). For example, the indicator FERST37 means the fluorescence intensity ratio of serum in the presence and absence of EvaGreen at 37°C.

The fluorescence intensities of the three groups are presented in Figure 1. Serum autofluorescence intensity and cfDNA-related fluorescence intensity were lower in HCC compared with BLD. All of the fluorescence indicators in the CH group and half of the fluorescence indicators in the LC group significantly differed from those in the HCC group.

### Correlations of serum fluorescence intensity with clinical data

The correlations of 6 florescence indicators with age, gender, AFP, ALT, AST, TBIL, DBIL, GGT, ALP, TP, ALB and GLB were analyzed. Of 72 correlation coefficients (absolute value), serum fluorescence indicators were not correlated with age, gender and GLB. Most of hepatic function test results were correlated with serum fluorescence indicators, of which serum TBIL and DBIL levels exhibit relatively higher correlation coefficients (0.257–0.451) compared with other indicators (0.114–0.270) (Table 2).

**Table 2:** Correlations of serum fluorescence indicators with clinical data in patients with AFP-elevated liver diseases

	Pearson correlation coefficient					
	FST8	FST37	FST8E	FST37E	FERST8	FERST37
Age	−0.023	0.038	−0.020	0.058	0.079	0.077
Gender^#^	−0.057	−0.071	−0.061	−0.067	0.025	0.028
AFP	−0.186^**^	−0.121^*^	−0.131^*^	−0.059	0.234^**^	0.234^**^
ALT	0.236^**^	0.153^**^	0.227^**^	0.128^*^	−0.189^**^	−0.194^**^
AST	0.246^**^	0.169^**^	0.270^**^	0.169^**^	−0.134^*^	−0.151^**^
TBIL	0.440^**^	0.257^**^	0.414^**^	0.209^**^	−0.384^**^	−0.364^**^
DBIL	0.451^**^	0.261^**^	0.426^**^	0.212^**^	−0.384^**^	−0.365^**^
GGT	0.152^**^	0.144^*^	0.205^**^	0.190^**^	−0.052	−0.039
ALP	0.194^**^	0.152^**^	0.228^**^	0.180^**^	−0.099	−0.074
TP	−0.134^*^	−0.090	−0.101	−0.066	0.147^**^	0.114^*^
ALB	−0.215^**^	−0.178^**^	−0.180^**^	−0.150^**^	0.210^**^	0.183^**^
GLB	0.029	0.048	0.037	0.052	−0.008	−0.024

^*^P < 0.05, ^**^P < 0.01. ^#^Kendall's tau-b correlation analysis. The names of fluorescence indicators are combinations of abbreviations representing the fluorescence intensity (F) of the serum sample (S) detected at a temperature of 8°C (T8) or 37°C (T37) in the presence (E) or absence of the dsDNA dye EvaGreen and their ratio (ER). AFP: alpha-fetoprotein; ALT: alanine aminotransaminase; AST: aspartate aminotransaminase; TBIL: total serum bilirubin; DBIL: direct serum bilirubin; GGT: gamma-glutamyl transferase; ALP: alkaline phosphatase; TP: total serum protein; ALB: serum albumin; GLB: serum gamma-globins.

### Diagnostic value of single indicators for differentiating HCC from BLD

All 6 serum fluorescence indicators had certain diagnostic value for differentiating HCC from BLD, with AUROCs of 0.615–0.801. Age, AFP and some items from hepatic function tests were also significant for differentiating HCC from BLD, with AUROCs of 0.574–0.790 (Table 3).

**Table 3:** AUROCs of single indicators for diagnosing HCC vs. BLD with elevated serum AFP levels

Indicator	AUROC (95% CI)
Serum fluorescence	
FST8	0.737 (0.680–0.794)^**^
FST37	0.687 (0.627–0.747)^**^
FST8E	0.665 (0.601–0.728)^**^
FST37E	0.615 (0.550–0.679)^**^
FERST8	0.801 (0.751–0.851)^**^
FERST37	0.782 (0.731–0.834)^**^
Age	0.669 (0.608–0.730)^**^
Gender	0.540 (0.474–0.607)
AFP	0.752 (0.700–0.804)^**^
Hepatic function tests	
ALT	0.763 (0.705–0.821)^**^
AST	0.644 (0.582–0.706)^**^
TBIL	0.783 (0.729–0.837)^**^
DBIL	0.790 (0.737–0.843)^**^
GGT	0.551 (0.489–0.614)
ALP	0.520 (0.457–0.583)
TP	0.576 (0.511–0.642)^*^
ALB	0.574 (0.509–0.639)^*^
GLB	0.543 (0.477–0.609)

^*^P < 0.05, ^**^P < 0.01. The names of fluorescence indicators are combinations of abbreviations representing the fluorescence intensity (F) of the serum sample (S) detected at a temperature of 8°C (T8) or 37°C (T37) in the presence (E) or absence of the dsDNA dye EvaGreen and their ratio (ER). HCC: hepatocellular carcinoma; BLD: benign liver disease; AFP: alpha-fetoprotein; ALT: alanine aminotransaminase; AST: aspartate aminotransaminase; TBIL: total serum bilirubin; DBIL: direct serum bilirubin; GGT: gamma-glutamyl transferase; ALP: alkaline phosphatase; TP: total serum protein; ALB: serum albumin; GLB: serum gamma-globins.

### The establishment and evaluation of diagnostic models

All continuous variables were transformed by natural logarithm (Ln). The subjects were randomly divided into the training set (approximately 70% of all cases) and the validation set (approximately 30% of all cases). The training set was used to establish diagnostic models for differentiating HCC from BLD by applying binary logistic stepwise regression analysis in which the covariates were indicators of serum fluorescence, AFP, hepatic function tests and age alone and in different combinations. Six models were established (detailed information presented in Table 4) and named by a combination of abbreviations representing the fluorescence intensity (F), AFP (P), hepatic function tests (H) and/or age (A) with “-M” (model), such as FPHA-M (the model established with the indicators of fluorescence intensity, AFP, hepatic function tests and age). All models were significant for discriminating HCC from BLD (*P* < 0.001). The model FPHA-M, which combined serum fluorescence with AFP, hepatic function tests and age (a total of 8 variables), was the best model fit for discriminating HCC from BLD.

**Table 4:** Diagnostic models established with different indicators for differentiating HCC from BLD with elevated serum AFP levels

Model	Function	Nagelkerke Pseudo R^2^	Likelihood ratio test	
			χ^2^	*P*
F-M	Logit(P) = −51.689 + 11.886LnFST37 – 7.173LnFST8 + 15.296LnFERST8	0.514	104.950	<0.001
P-M	Logit(P) = −1.762 + 0.508LnAFP	0.360	41.974	<0.001
H-M	Logit(P) = −0.549 – 1.035LnALT – 1.443LnDBIL + 1.927 LnALP	0.545	113.347	<0.001
FP-M	Logit(P) = −48.146 + 12.641LnFST37 – 8.383LnFST8E + 22.399LnFERST8 + 0.532LnAFP	0.607	130.907	<0.001
FH-M	Logit(P) = −71.499 + 6.175LnFST37 + 17.525LnFERST8 – 1.268LnALT – 1.300LnDBIL + 1.797LnALP	0.690	156.596	<0.001
FPHA-M	Logit(P) = −100.861 + 6.697LnFST37 + 18.269LnFERSET8 + 3.501LnAge – 1.196LnALT + 5.045LnTBIL – 6.103LnDBIL + 2.227LnALP + 0.879LnAFP	0.803	197.244	<0.001

All indicators were transformed by natural logarithm (Ln). Each model name is a combination of abbreviations representing the fluorescence intensity (F), alpha-fetoprotein (P), hepatic function tests (H) and/or age (A) with “-M” (model), indicating the covariates used during modeling. For example, FPHA-M was established using the indicators of fluorescence intensity, alpha-fetoprotein, hepatic function tests and age. The fluorescence indicators represent the fluorescence intensity (F) of a serum (S) sample at a detection temperature of 8°C (T8) or 37°C (T37) in the presence (E) or absence of the dsDNA dye EvaGreen or their ratio (ER). HCC: hepatocellular carcinoma; BLD: benign liver disease; AFP: alpha-fetoprotein; ALT: alanine aminotransaminase; TBIL: total serum bilirubin; DBIL: direct serum bilirubin; ALP: alkaline phosphatase.

**Figure 2:**
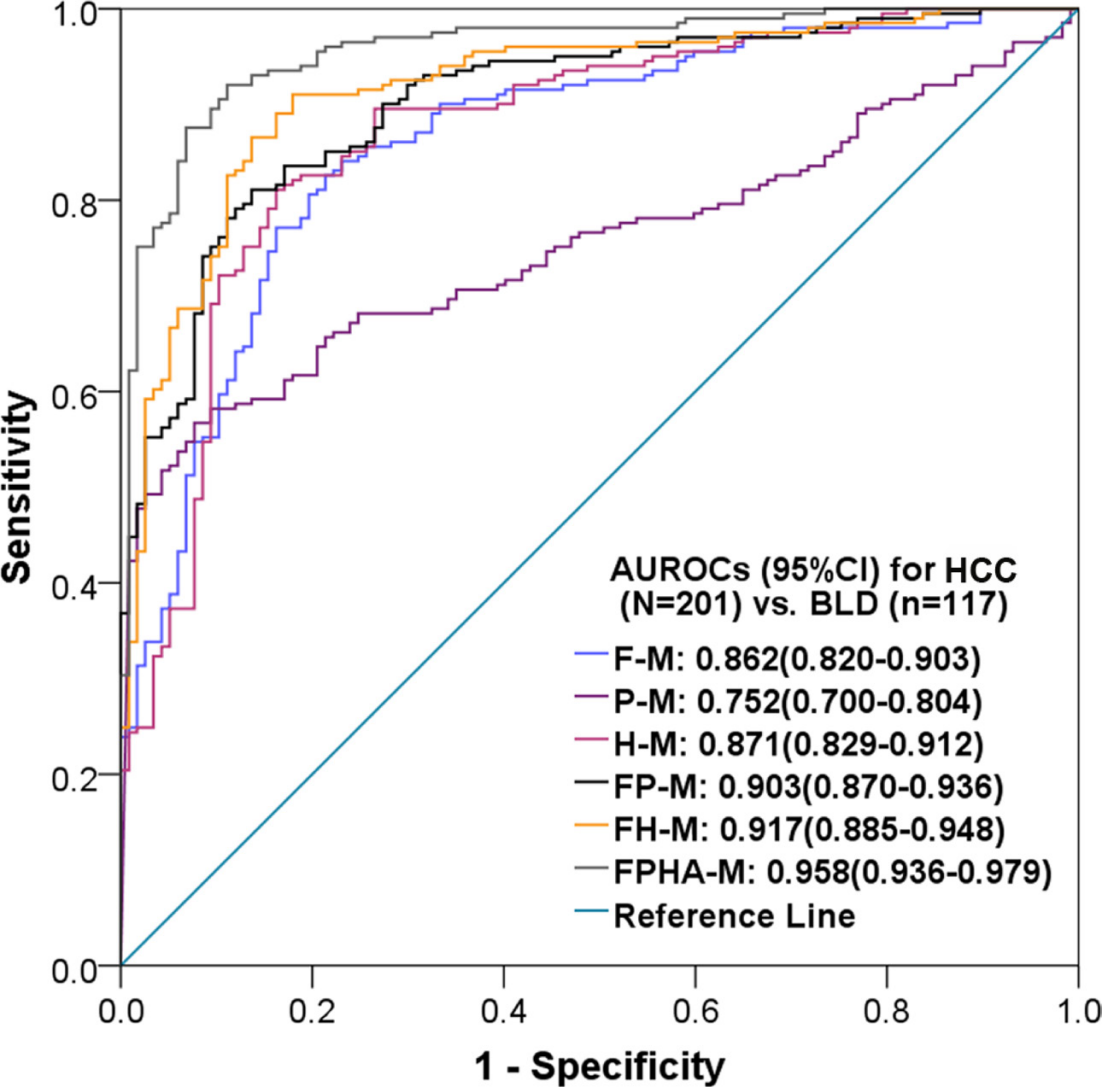
ROC curves and AUROCs of six diagnostic models for diagnosing HCC vs. BLD with elevated serum AFP levels AUROC: area under the receiver operating characteristic curve; CI: confidence interval; HCC: hepatocellular carcinoma; BLD: benign liver disease. Each model name is a combination of abbreviations representing fluorescence intensity (F), alpha-fetoprotein (P), hepatic function tests (H) and/or age (A) with “-M” (model), which indicates the related covariates used during modeling. For example, FPHA-M was established with indicators of fluorescence intensity, alpha-fetoprotein, hepatic function tests and age.

The AUROCs and diagnostic performances of the six models in the training set, validation set and complete set are presented in Table 5. The model established exclusively with AFP was fair for discriminating HCC from BLD (AUROC <0.8). The models established exclusively with fluorescence intensity indicators or hepatic function tests were good for discriminating HCC from BLD (AUROC >0.8). The models established with multiple indicators improved the diagnostic value for HCC. The diagnostic performances of the FPHA-M model in training set, validation set and complete set were excellent in discriminating HCC from BLD (AUROC ≥0.94). The AUROCs and accuracies of these models were similar among training sets, validation sets and complete sets. The ROC curves and AUROCs of the six models for differentiating HCC from BLD in the complete set are presented in Figure 2.

**Table 5:** Diagnostic performances of six models for differentiating HCC from BLD with elevation of serum AFP

Model	AUROC (95% CI)	SEN (%)	SPE (%)	ACC (%)	PPV (%)	NPV (%)	PLR	NLR
F-M								
Training set	0.878 (0.830–0.925)	85.6	79.5	83.3	87.5	76.7	4.18	0.18
Test set	0.849 (0.771–0.927)	83.9	76.5	81.3	86.7	72.2	3.56	0.21
Complete set	0.862 (0.820–0.903)	82.6	78.6	81.1	86.9	72.4	3.87	0.22
P-M								
Training set	0.743 (0.580–0.806)	54.7	**92.8**	68.9	**92.7**	55.0	7.56	0.49
Test set	0.776 (0.685–0.868)	62.9	**91.2**	72.9	**92.9**	57.4	7.13	0.41
Complete set	0.752 (0.700–0.804)	56.7	**92.3**	69.8	**92.7**	55.4	7.37	0.47
H-M								
Training set	0.883 (0.836–0.931)	84.9	81.9	83.8	88.7	76.4	4.70	0.18
Test set	0.850 (0.770–0.930)	82.3	82.4	82.3	89.5	71.8	4.66	0.22
Complete set	0.871 (0.829–0.912)	81.1	83.8	82.1	89.6	72.1	4.99	0.23
FP-M								
Training set	0.913 (0.876–0.951)	82.0	88.0	84.2	**91.9**	74.5	6.81	0.20
Test set	0.886 (0.820–0.952)	79.0	85.3	81.3	**90.7**	69.0	5.37	0.25
Complete set	0.903 (0.870–0.936)	81.1	86.3	83.0	**91.1**	72.7	5.93	0.22
FH-M								
Training set	0.936 (0.904–0.969)	87.1	89.2	87.8	**93.1**	80.4	8.03	0.15
Test set	0.884 (0.815–0.953)	82.3	88.2	84.4	**92.7**	73.2	6.99	0.20
Complete set	0.917 (0.885–0.948)	86.6	86.3	86.5	**91.6**	78.9	6.33	0.16
FPHA-M								
Training set	0.970 (0.951–0.988)	**92.1**	**92.8**	**92.3**	**95.5**	87.5	**12.74**	0.09
Test set	0.940 (0.887–0.992)	**95.2**	85.3	**91.7**	**92.2**	**90.6**	6.47	0.06
Complete set	0.958 (0.936–0.979)	**92.0**	88.9	**90.9**	**93.4**	86.7	8.28	0.09

The training set includes 139 cases in the hepatocellular carcinoma group and 83 cases in the benign liver diseases. The test set includes 62 hepatocellular carcinoma cases and 34 benign liver disease cases. Each model name is a combination of abbreviations representing the fluorescence intensity (F), alpha-fetoprotein (P), hepatic function tests (H) and/or age (A) with “-M” (model), indicating the related covariates used during modeling. For example, FPHA-M was established with the indicators of fluorescence intensity, alpha-fetoprotein, hepatic function tests and age. HCC: hepatocellular carcinoma; BLD: benign liver disease; AUROC: area under the receiver operating characteristic curve; CI: confidence interval; SEN: sensitivity; SPE: specificity; ACC: accuracy; PPV: positive predictive value; NPV: negative predictive value; PLR: positive likelihood ratio; NLR: negative likelihood ratio.

### Positive rates of the diagnostic models in HCC and BLD patients

We calculated positive rates of the F-M, P-M, H-M and FPHA-M models in HCC and BLD patients with different levels of elevated serum AFP, clinical stages and tumor sizes (Table 6). F-M, H-M and FPHA-M models exhibited significantly increased positive rates than P-M for HCC, including different levels of elevated serum AFP, BCLC stages and tumor sizes. In the groups with different levels of elevated serum AFP, the F-M, H-M and FPHA-M models were more valuable for diagnosing low-level AFP-elevated HCC compared with P-M but with increased false positive rates. Of the four models, FPHA-M exhibits significantly increased diagnostic positive rates for HCC compared with other models.

**Table 6:** Positive rates of the models in serum AFP-elevated patients with HCC or BLD

	Positive/total cases (%)							
	F-M		P-M		H-M		FPHA-M	
	HCC	BLD	HCC	BLD	HCC	BLD	HCC	BLD
AFP levels								
7- < 20.0	**36/42 (85.7)**	12/46 (26.1)	0/42 (0.0)	0/46 (0.0)	**37/42 (88.1)**	11/46 (23.9)	31/42 (73.8)	3/46 (6.5)
20~< 200	30/39 (76.9)	9/51 (17.6)	0/39 (0.0)	0/51 (0.0)	29/39 (74.7)	7/51 (13.7)	31/39 (79.5)	2/51 (3.9)
200~< 400	**6/7 (85.7)**	2/11 (18.2)	1/7 (14.3)	0/11 (0.0)	5/7 (71.4)	1/11 (9.1)	**7/7 (100.0)**	1/11 (9.1)
≥400	90/113 (79.6)	0/9 (0.0)	**113/113 (100.0)**	**9/9 (100.0)**	**93/113 (82.3)**	1/9 (11.1)	**107/113 (94.7)**	2/9 (22.2)
Total	**162/201 (80.6)**	23/117 (19.7)	114/201 (56.7)	9/117 (7.7)	**164/201 (81.6)**	20/117 (17.1)	**176/201 (87.6)**	8/117 (6.8)
BCLC stage								
A	37/49 (75.5)	-	21/49 (42.9)	-	**43/49 (87.8)**	-	**40/49 (81.6)**	-
B	81/103 (78.6)	-	60/103 (58.3)	-	82/103 (79.6)	-	**92/103 (89.3)**	-
C&D	**44/49 (89.8)**	-	33/49 (67.3)	-	39/49 (79.6)	-	**44/49 (89.8)**	-
Total	**162/201 (80.6)**	-	114/201 (56.7)	-	**164/201 (81.6)**	-	**176/201 (87.6)**	-
Tumor size								
≤3 cm	27/35 (77.1)	-	14/35 (40.0)	-	**29/35 (82.9)**	-	26/35 (74.3)	-
3.1–5 cm	17/22 (77.3)	-	9/22 (40.9)	-	**18/22 (81.8)**	-	**20/22 (90.9)**	-
>5 cm	**95/116 (81.9)**	-	73/116 (62.9)	-	**95/116 (81.9)**	-	**105/116 (90.5)**	-
Total	**139/173 (80.3)**		96/173 (55.5)	-	**142/173 (82.1)**	-	**151/173 (87.3)**	-

Each model name is a combination of abbreviations representing the fluorescence intensity (F), alpha-fetoprotein (P), hepatic function tests (H) and/or age (A) with “-M” (model), indicating the related covariates used during modeling. For example, FPHA-M was established with the indicators of fluorescence intensity, alpha-fetoprotein, hepatic function tests and age. HCC: hepatocellular carcinoma; BLD: benign liver diseases.

## DISCUSSION

In the present study, we developed a method for identifying HCC in liver diseases with elevated AFP levels. We collected serum specimens and clinical data from a group of patients with HCC, LC or CH whose serum AFP levels were elevated. Serum autofluorescence and cfDNA-related fluorescence intensities were measured in these patients. The values of serum fluorescence indicators, AFP, hepatic function tests and age were evaluated alone and in combination for differentiating HCC from BLD. Several diagnostic models established based on combinations of different data above were valuable in differentiating HCC from BLD, especially the model established with serum fluorescence, AFP, hepatic function tests and age, which exhibited excellent performance for differentiating HCC from BLD with elevated serum AFP levels.

The serum autofluorescence intensities were significantly weaker in HCC compared with BLD, with fair differentiation powers (AUROC = 0.687–0.737). Serum autofluorescence is produced by endogenous fluorescent substances, including proteins, lipids, retinoids, lipofuscins, lipofuscin-like lipopigments, ceroids [15] and bilirubin [16]. Enhanced fluorescence intensity is observed when bilirubin binds to albumin [17]. Serum autofluorescence intensities mainly correlated with bilirubin levels in the present results are consistent with the above reports. However, the correlation coefficients were only approximately 0.4, and the autofluorescence indicator and billirubin were concurrently entered into the diagnostic models established by logistic stepwise regression analysis. These results suggest that serum autofluorescence is an independent factor for differentiating HCC from BLD and naturally fluorescent substances in the serum differ between HCC and BLD.

Conversely, cfDNA-related fluorescence intensities were significantly stronger in HCC compared with BLD in the present study. The fluorescence indicators FERST8 and FERST37, which reflect cfDNA-related fluorescence, were significantly increased in HCC compared with BLD and exhibited higher AUROCs compared with autofluorescence indicators (FST8 and FST37) for differentiating HCC from BLD, indicating that the amount of serum cfDNA in the HCC group is increased compared with the BLD group. These results are consistent with previous reports by other groups, in which the levels of serum cfDNA were significantly increased in the sera from HCC patients compared with sera from non-cancer patients [13, 18, 19]. Compared with these studies, we detected the cfDNA-related fluorescence intensity directly using the nucleic acid dye EvaGreen in a real-time PCR system instead of PCR amplification or other complex methods. Our system offers the advantages of convenience and low cost and is thus more practical.

Although the cfDNA-related fluorescence indicator FERST8 had the maximum AUROC (0.801) for differentiating HCC from BLD in all single indicators, including serum fluorescence intensity, AFP and hepatic function tests, this indicator is not ideal in clinical application. Generally, a single biomarker, including traditional biomarkers and new biomarkers of liver cancer, is insufficient for the diagnosis of HCC in sensitivity and/or specificity [20]. To overcome the limitation of a single indicator in diagnosis, a panel of biomarkers is typically applied to improve diagnostic value for discriminating HCC from BLD [21–23]. However, the traditional biomarker panel has at least two obvious defects: low-throughput detection (separately detected) and inverse variation of sensitivity and specificity. The integration of high-throughput detection and multivariate analysis is an ideal strategy to effectively address the above defects. Zhang *et al* [24] screened candidate miRNAs by microarray analysis and identified a 3-miRNA panel (miR-92-3p, miR-107, and miR-3126-5p) by logistic regression analysis that was valuable for diagnosing early stage HCC and low-level AFP HCC. In the present study, high-throughput and simple measurements of two types of serum fluorescence intensity (serum autofluorescence and cfDNA-related fluorescence) followed by multivariate analyses offer the advantages of convenience and efficacy for diagnostic application of serum fluorescence.

In this study, we found that some factors from hepatic function tests were valuable for differentiating HCC from BLD and comparable to AFP. The diagnostic model that combined items from hepatic function tests exhibited good differentiation power and was superior to AFP. In clinical practice, the value of general data is typically ignored in the diagnosis of HCC as any these single items are not sufficiently valuable for diagnostic application. In fact, the patient's general data in combination are valuable for the diagnosis of HCC. Best *et al* [25] combined gender and age with three biomarkers (AFP, AFP-L3 and DCP) to establish a diagnostic score system that exhibited the highest AUROC for diagnosing early stage HCC. The system was superior to the combinations of the three markers alone. We previously combined conventional blood tests with serum fluorescence by logistic models and achieved excellent diagnostic performances for early, small and AFP-negative PHC [14]. In the present study, the diagnostic model FPHA-M established with indicators of serum fluorescence intensity, AFP, hepatic function tests and age exhibited the best performance for differentiating HCC from BLD among all models of combinations of different indicators. Therefore, we should not neglect the combination of general clinical data with specific indicators for diagnostic application, although the diagnostic power of each factor of general clinical data may be not strong enough.

We calculated the positive rates of four main diagnostic models in two groups of patients and found that the AFP model (P-M) was not valuable for the differentiation of HCC from BLD because HCC or BLD patients were all positive when serum AFP ≥400 ng/ml and almost negative when serum AFP <400 ng/ml, suggesting that serum AFP levels lack value in discriminating HCC from BLD with elevated serum AFP levels. In contrast, the FPHA-M model exhibited a considerably increased positive rate compared with the AFP model (87.6% vs 56.7%) in total HCC and a comparable low false positive rate (6.8% vs 7.7%) in BLD. In addition, considerably increased positive rates were similarly noted compared with the AFP model in early (81.6% vs. 42.9%), small (74.3% vs. 40.0%) and AFP-negative (73.8% vs. 0%) HCCs. These results indicate that the FPHA-M model is robust for differentiating HCC from BLD with increased AFP levels, including early, small and AFP-negative HCCs.

Although the method we developed in the present study is useful for identifying HCC in liver diseases with increases in serum AFP, it should be evaluated and confirmed by multicenter studies with a larger sample size. Additionally, the subjects in this study were in-patients; thus, the value of this method for identifying out-patients with HCC among liver diseases with elevated serum AFP levels is unknown.

In conclusion, we developed a simple but powerful method to identify HCC among liver diseases with serum AFP levels greater than 7 ng/ml. In this method, dual serum fluorescence (autofluorescence and cfDNA-related fluorescence) was conveniently measured using a conventional real-time PCR system, offering the advantages of high-throughput, speed and low cost. The diagnostic model established with the indicators of both types of fluorescence intensity presents better performance than AFP for differentiating HCC from BLD, and the diagnostic model that combined the fluorescence indicators, AFP, hepatic function tests and age exhibited excellent performance for differentiating HCC from BLD, with an AUROC greater than 0.95 and an accuracy more than 90%. To our knowledge, this is the first report to apply dual serum fluorescence alone and combined with general clinical data to identify HCC in liver diseases with elevated serum AFP levels. However, a multicenter study with a larger sample size and scope of subjects should be performed for the further evaluation and possible optimization of this method.

## MATERIALS AND METHODS

### Collection of serum specimens and clinical data

Leftover serum specimens (initially drawn for routine laboratory blood tests) of patients with HCC, LC or CH prior to therapy were collected from the First Affiliated Hospital of Nanchang University, and patients with abnormal serum AFP levels (>7 ng/ml) were selected as subjects. The clinical data of these patients were collected, including age, gender, serum AFP levels, hepatic function tests [alanine aminotransaminase (ALT), aspartate aminotransaminase (AST), total serum bilirubin (TBIL), direct serum bilirubin (DBIL), gamma-glutamyl transferase (GGT), alkaline phosphatase (ALP), total serum protein (TP), serum albumin (ALB) and serum gamma-globins (GLB)], medical imaging and pathology (if available). HCC was diagnosed based on pathology or noninvasive diagnostic criteria (coincidental results of B type ultrasound and CT and/or MRI) [26]. LC and CH were diagnosed based on clinical manifestation, laboratory tests and liver medical imaging. This study was approved by the First Affiliated Hospital of Nanchang University Committee for Clinical Investigation, which determined that patient consent was not necessary.

### Fluorescence intensity measurement of serum specimens

**Figure 3:**
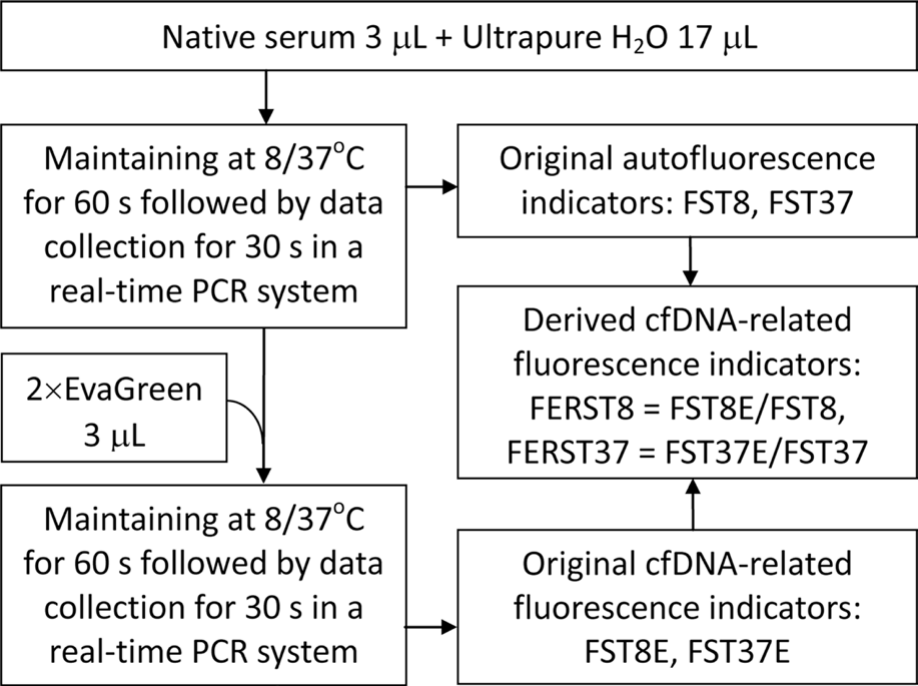
Diagram of the measurement of serum autofluorescence and cell-free DNA-related fluorescence and the fluorescence indicators The names of fluorescence indicators are abbreviations of the fluorescence intensity (F) of the serum (S) sample at a given temperature (T) of 8°C (8) or 37°C (37) in the presence (E) or absence of the dsDNA dye EvaGreen and their ratio (ER).

The autofluorescence intensity and cfDNA-related fluorescence intensity of serum specimens were measured by the method that we previously established [14] and simplified in this study (Figure 3), which was performed in a real-time PCR system at the excitation and emission wavelengths of 490 nm and 525 nm, respectively. Six fluorescence indicators were obtained after measurement, including 2 original serum autofluorescence indicators (FST8 and FST37), 2 original cfDNA-related fluorescence indicators (FST8E and FST37E) and 2 derived cfDNA-related fluorescence indicators (FERST8 and FERST37). These serum fluorescence indicators were named based on a combination of several abbreviations: F (fluorescence), S (serum), T (temperature), E (EvaGreen), 8 (8°C), 37 (37°C) and R (ratio), indicating the serum fluorescence intensity measured under a specific condition. For example, FERST37 indicates the fluorescence intensity ratio for serum in the presence and absence of EvaGreen at 37°C.

### Statistical analyses and diagnostic performance evaluation

All statistical analyses were performed using SPSS Statistics 20.0 (IBM, Armonk, NY, USA). Continuous variables were expressed as the mean and standard deviation (mean ± SD) and compared among groups using a one-way ANOVA. Categorical variables were expressed as a frequency or percentage and compared using the Pearson's Chi-squared test or Fisher's exact test. Correlations among variables were analyzed with Pearson or Kendall's tau-b correlation analysis. All statistical tests were two-sided, and *P* < 0.05 indicated significant difference.

The binary logistic stepwise regression analysis was used to establish diagnostic models for differentiating HCC from BLD. The “estimated response probabilities” of models were saved during logistic regressions and used as the test variables in the ROC curve analyses. The point with the largest Youden's index in the “coordinate points of ROC curve” was selected as the cut-off value to calculate diagnostic performance (sensitivity, specificity, accuracy, positive/negative predictive value, and positive/negative likelihood ratio).

## Abbreviations

AFP: alpha-fetoprotein
ALB: serum albumin
ALP: alkaline phosphatase
ALT: alanine aminotransferase
AST: aspartate aminotransaminase
AUROC: area under the receiver operating characteristic curve
BLD: benign liver disease
cfDNA: cell-free circulating DNA
CH: chronic hepatitis
CI: confidence interval
DBIL: direct serum bilirubin
GGT: gamma-glutamyl transferase
GLB: serum gamma-globins
HCC: hepatocellular carcinoma
LC: liver cirrhosis
ROC: the receiver operating characteristic curve
TBIL: total serum bilirubin
TP: total serum protein.
